# Few-Shot Object Detection: Application to Medieval Musicological Studies

**DOI:** 10.3390/jimaging8020018

**Published:** 2022-01-19

**Authors:** Bekkouch Imad Eddine Ibrahim, Victoria Eyharabide, Valérie Le Page, Frédéric Billiet

**Affiliations:** 1 Sorbonne Center for Artificial Intelligence, Sorbonne University, 75005 Paris, France; 2 STIH Laboratory, Sorbonne University, 75005 Paris, France; maria-victoria.eyharabide@sorbonne-universite.fr; 3 IReMus Laboratory, Sorbonne University, 75002 Paris, France; egapel@gmail.com (V.L.P.); frederic.billiet@sorbonne-universite.fr (F.B.)

**Keywords:** transfer learning, few-shot image classification, few-shot object detection, cultural heritage, musical iconography, medieval singing

## Abstract

Detecting objects with a small representation in images is a challenging task, especially when the style of the images is very different from recent photos, which is the case for cultural heritage datasets. This problem is commonly known as few-shot object detection and is still a new field of research. This article presents a simple and effective method for black box few-shot object detection that works with all the current state-of-the-art object detection models. We also present a new dataset called MMSD for medieval musicological studies that contains five classes and 693 samples, manually annotated by a group of musicology experts. Due to the significant diversity of styles and considerable disparities between the artistic representations of the objects, our dataset is more challenging than the current standards. We evaluate our method on YOLOv4 (m/s), (Mask/Faster) RCNN, and ViT/Swin-t. We present two methods of benchmarking these models based on the overall data size and the worst-case scenario for object detection. The experimental results show that our method always improves object detector results compared to traditional transfer learning, regardless of the underlying architecture.

## 1. Introduction

The analysis of medieval vocal practices is an essential issue for musicologists and performers. However, medieval singing as a musical performance has been explored much less than other musical instrumental performances [1]. This is because medieval musical instruments are studied mainly by analyzing images of artworks in which these instruments are represented [2]. However, since the human vocal cords cannot be displayed explicitly, it is harder to identify whether a person or group is singing or not.

Even though there are numerous descriptions of musicians and singers in medieval chronicles and tales, illuminated manuscripts are the principal source for musical iconography. Illuminations often depict very complex situations in a tiny space. Artists often wished to concentrate much more information in a small illumination than would be contained withinthat scene in real life. However, studying a large corpus of images allows musicologists to detect repeated patternsand shed light on previously unknown medieval vocal practices across different periods and regions. The discovered patterns could enable performers wishing to perform repertoires to better understand the organization of singers, the environment, and the setting of the songs according to the period and genre considered. For example, considering the architectural modifications over the centuries, better choices regarding locations and musicians could be made to recreate acoustics as close as possible to the original music scene. Therefore, our objective is to find singing performances in images from medieval artworks. More precisely, we will detect medieval images containing persons in solo or group-singing situations, whether accompanied or not by musical instruments. The final objective for musicologists is to better understand the physical postures of singers, their relationship, and their location inside the building.

**Figure 1:**
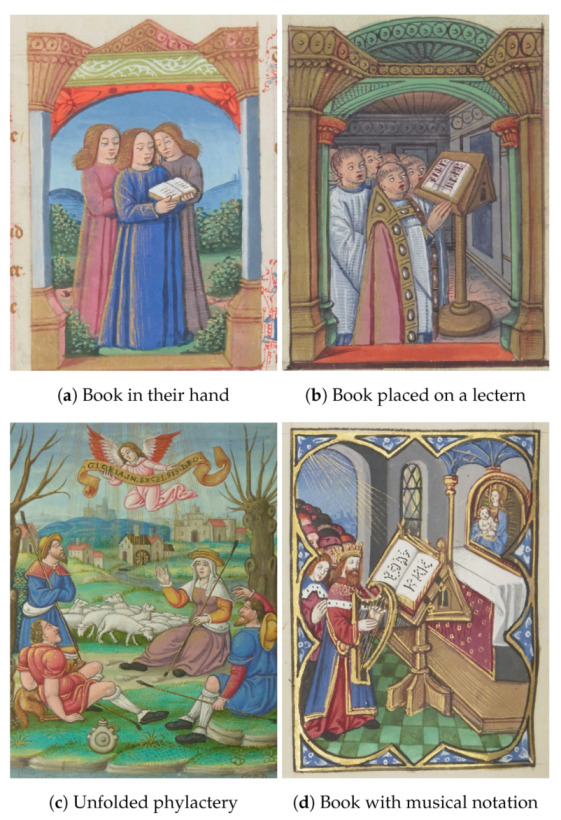
Examples of medieval singing illuminations.

Since the human voice is not a visible musical instrument, it is necessary to define possible objects in the images that may suggest the presence of singing performances. Therefore, we propose identifying characters who have their mouths open, perhaps with features linked to the vocal utterance (such as declamation or singing; see Figure 1a (BnF ms. fr. 166 f. 121v: https://gallica.bnf.fr/iiif/ark:/12148/btv1b105325870/f256/1500,750,1000,1300/full/0/native.jpg, accessed on 1 November 2021)). However, having the mouth open is not a sufficient condition to determine that a person is singing. The context or environment in which these singers are performing is vital to understanding the musical scene.

The scene’s context should be analyzed to detect additional clues such as a book hold in the hands or knees, or placed on a lectern (Figure 1b (BnF ms. fr. 166 f. 115: https://gallica.bnf.fr/iiif/ark:/12148/btv1b105325870/f243/2799,3000,1000,1200/full/0/native.jpg, accessed on 1 November 2021)), an unfolded phylactery (and if visible, the text on the phylactery, Figure 1c (BnF. NAL 104, f. 50r: https://gallica.bnf.fr/iiif/ark:/12148/btv1b10023007f/f106/50,50,2000,2300/full/0/native.jpg, accessed on 1 November 2021)), or some musical notation (Figure 1d (BnF ms. fr. 166 f. 126v: https://gallica.bnf.fr/iiif/ark:/12148/btv1b105325870/f266/3199,3100,1000,1200/full/0/native.jpg, accessed on 1 November 2021)). Moreover, some gestures such as the hand placed on the shoulder, the movement of the pulse (to set the tempo and anchor the rhythm), or a finger pointing to the musical score may also evoke singing performances. Musicologists also analyze the texts embellished by the miniatures or illuminations containing singing performances. This research deliberately sets aside animals, hybrids, and monsters to concentrate only on clerics, laity, children, and angels.

Object detection is one of the most important tools in computer vision for musicologists and art historians working on cultural heritage (CH) data [1,3,4]. However, cultural heritage represents an interesting challenge for object detection since the data are mainly characterized by (i) small regions of interest; (ii) artistic creativity, leading to significant visual differences between instances of the same class; and especially (iii) a lack of properly annotated data for training. Regarding the last drawback, even though cultural institutions constantly publish raw datasets, the available images are domain-dependent and differ considerably depending on the material used, the technique used, and the artwork’s time [1]. Most of the previously mentioned challenges can be approached with object detection or a variant of it, such as its use in combination with domain adaptation techniques or visual transformers [5].

When dealing with small datasets (such as those for medival studies) that are challenging even for typical transfer learning methods, few-shot image classification is a possible solution [6]. Few-shot object detection is the process of learning and generalizing useful computer vision models from a small set of images [7]. The technique of few-shot learning [8] in computer vision has progressed drastically over the last years, mainly due to the advances in transfer learning techniques such as meta-learning [9]. Such advances have provided great results for basic image classification tasks. Our datasets in the field of cultural heritage suffer from a great deal of challenges, as described in [2], but the main challenges are the lack of samples in specific classes due to the loss of historical artifacts or the difficulty of finding such samples in the vast collections provided by different museums. Unlike image classification [10], few-shot object detection has received far less attention in the past and is still a growing field. The main difference between image classification and object detection is that the model is required to detect the location of the classified objects from a possible set of millions of potential locations. This additional sub-task makes the object detection task even harder to perform in scenarios where annotated data are sparse.

In this article, we present two main contributions, (i) a novel technique for performing few-shot object detection based on bi-stage training, in which the first stage tries to improve on the object localization process for the new classes and the second stage aims to improve the image classification and fine tuning of the pre-located coordinates; and (ii) a benchmark for three main models in the field of object detection, which are Yolov4 [11], Faster RCNN [12], and SWIN Transformers [13]. We chose these three architectures because they represent the leading representatives of their family trees of architectures, which are the RCNN family [14,15,16], the YOLO family [17], and the Visual Transformers family [5].

The paper is organized as follows: Section 2 summarizes the current state of the art in few-shot object detection and its applications to the domain of cultural heritage. Section 3 focus on presenting the dataset collection and annotation processes. Section 4 presents our novel and simple method for few-shot object detection. The empirical benchmark of our algorithm is shown in Section 5. Finally, Section 6 summarizes the contributions of the paper.

## 2. Related Works

### 2.1. Object Detection

Object detection, as a subtask of computer vision, has been the focus of tremendous research interest over the past few years, from traditional computer vision algorithms such as Viola Jones, and the Histogram of Oriented Gradients Detector, which are still commonly used in mobile applications for their speed and accuracy, to new deep-learning-based models [18,19,20] such as Yolo [17], RCNN [16], SSD [21], and others. We can split the deep learning-based object detection models into two subgroups: one-stage detectors and two-stage detectors. One-stage detectors are famous for their speed, which started with the Yolo Tree family (from v1, v2, v3, which are the original models up to v4, v5, and pp, which are extensions provided by separate researchers). Other one-stage detectors were introduced in the field, such as the Single-Shot MultiBox Detector (SSD), and RetinaNet [22]. The Single-Shot MultiBox Detector (SSD) introduced the multi-reference and multi-resolution detection techniques, allowing better accuracy. The developers of RetinaNet argued that the single-shot detectors have low accuracy because of the imbalance between background and foreground classes and introduced the focal loss so that the detector would focus more on challenging examples throughout the training phase.

The second category of models is two-stage detectors, which are known for their accuracy but lack speed compared with single-shot detectors. Two-stage classifiers are very useful in cases where the inference time is not crucial, and there is no need for fast or real-time processing. Such cases are widespread in cultural heritage studies or medical applications, where accuracy is more important than increasing speed by a few microseconds. The branch of two-stage detectors started with the introduction of region-based convolutional neural networks (RCNNs), which combined deep learning with the traditional selective search algorithm, and which were later abandoned due to the development of faster RCNN models propoingd a fully deep approach based on region proposal networks. Although RCNN-based models have dominated this side of the family tree of object detection, especially with the introduction of feature pyramid networks, other models have also been proposed for two-stage detectors, such as spatial pyramid pooling networks (SPPNet) [23], which demonstrated many ideas that later found their way into the RCNN family.

All the previously mentioned deep models are convolution-based, mainly because CNNs have dominated the field of computer vision due to their performance as of 2021. Lately, however, a new branch of models is being added to the computer vision field, which are attention-based models, rather than CNN-based models. Attention is an idea that has been dominating the field of natural language processing for a long time now, with models such as bert and GPT, which provide a human-like level of understanding of text and responding to questions. This trend has found its way to computer vision thanks to the paper “An Image is Worth 16 × 16 Words”, demonstrating an approach commonly known as vision transformer (ViT) [5], as well as its follow-ups which applied a transformer architecture on 16 × 16 non-overlapping medium-sized image patches for image classification. Although ViT provided a good speed-accuracy trade-off compared with CNN-based models, its successful application required a large-scale dataset and training, which was later fixed using data-efficient image transformers (DeiT) [24], which proposed several training algorithms allowing the vision transformers to be applied on smaller datasets. Vision transformers are aiming to replace CNNs and outperform them in terms of speed and accuracy, and the best example of this is the current state-of-the-art model for image classification and object detection/instance segmentation, the Swin Transformer [13], which builds a hierarchical vision transformer using shifted windows that can be used as a generic backbone for any computer vision model, replacing the convolutional layers and outperforming them with a large gap in terms of performance metrics such as top-1 accuracy and mean average precision (mAP) [25], and which has linear computational complexity with respect to the input image size.

### 2.2. Few-Shot Object Detection

Few-shot object detection has been a growing field lately but has not received as much attention as object detection for large-scale datasets or even few-shot image classification, mainly due to the task’s difficulty compared with image classification and the large variability of the models’ architecture in the object detection field [26]. Nonetheless, several proposals have been used in the field, such as meta-learning-based techniques [27], feature re-weighting [6] and fine-tuning-based approaches, such as the frustratingly simple few-shot object detection [7] method, which is very similar to our method but which lacks its flexibility and applicability to other architectures.

### 2.3. Computer Vision in the Humanities

Although the field of object detection is relatively mature and has been around for quite some time, its applications to cultural heritage data have been relatively modest. In the musicology field, most contributions use simple images [28], such as the recent contributions to digital cultural heritage analysis focusing on similarity metric learning methods for making semantic-level judgments, such as predicting a painting’s style, genre, and artist [29,30]. Other contributions detect fake artworks through stroke analysis and an artistic style transfer using adversarial networks to regularize the generation of stylized images [31]. Other applications of deep networks to archaeological remote sensing include topics such as the detection of buried sites on Arc GIS data [32] and the classification of sub-surface sites using R-CNNs on LiDAR data [33]. Both contributions followed a transfer learning approach by fine-tuning a pre-trained CNN using LiDAR data in ImageNet [34]. Overall, we can see that the application of computer vision in the digital humanities and cultural heritage is a field that is still being uncovered, mainly because of the lack of data, and this why is our method for few-shot object detection will open a door towards more contributions in the field, overcoming the barrier of the lack of data.

## 3. Dataset Annotation and Ground Truth Creation

The image dataset creation and the ground truth annotation and validation processes were organized into four different steps, described as follows.

1. image dataset: The first step was to create a dataset of images for training. Therefore, three experts in musicology and professional singers searched for and manually selected images of manuscript pages containing singing representations.
2. Annotate objects: The domain experts annotated the dataset using the Supervisely tool (https://supervise.ly/, accessed on 1 November 2021), which is a collaborative online tool for image annotation, allowing users to create bounding boxes and object masks. As a result, the objects (such as books, lecterns, altars) in each image are highlighted by defining its borders.
3. Classify the objects: In the third step, the objects annotated previously were manually classified as book, folio, phylactery, lectern, or altar by the musicologists. Thus, we can not only detect objects but also the exact position of those objects within the image.
4. Obtain a consensus in the classification of objects: As we explained previously, it is not easy to detect singing performances. We are working with images of artworks, so the singing representations are not real; they are paintings or drawings of an artist who does not necessarily know about vocal practices. Therefore, the fourth and last step in the classification of objects consists of achieving a consensus among all the experts to create the ground truth.

### 3.1. Image Dataset of Illuminations Representing Medieval Singing

At the beginning of the Middle Ages, musical instruments were mainly used as a complement for singing. At that time, in religious music, the vocal song was thought to represent the divine Word of God. Therefore, religious music was promoted and developed by high authorities of the Church. Medieval singing was present in all services, major festivals, and ceremonies. Religious songs were repeated every day in churches and monasteries. In secular music, singing performances werealso of vital importance. Secular vocal songs were generally transmitted orally. They reflected ordinary people’s daily lives, love and war stories, or songs intended for processions going to battle.

The importance of the notation of songs is also manifested by rich illuminations, which gradually range from simple colored to intricately decorated capitals and complex scenes with multiple characters and rich details. These richly illuminated manuscripts provide musicologists with numerous clues concerning the practice of singing. Analyzing a large dataset of images may reveal previously unknown details to clarify medieval singing beyond eras and regions.

Massive amounts of digital iconographic data are available nowadays thanks to the development of the IIIF standard. The IIIF standard offers unified access to view and read digitized ancient documents, significantly increasing the number of people who can consult them. The most important cultural institutions all around the world publish their collections using the IIIF format. The vast digitization of manuscripts now makes it possible to reconsider iconographic studies and analyze a series of gestures and behaviors that can be compared with narratives, descriptions, and texts of treatises. Thus, new illuminated medieval collections are now available for researchers, allowing them to harvest millions of illuminated manuscripts. The ever-increasing number of singing performances hidden in those illuminated manuscripts challenges researchers to develop new pattern-recognition methods to find them.

Our musicologists visualized hundreds of IIIF manuscripts from various collections to find images of illuminations with singing performances. Among the consulted institutions were the French National Library (BnF), the J. Paul Getty Museum, the Universitatsbibliothek Basel, the University of Cambridge, and the University of Princeton. As a result, we obtained a dataset of 341 IIIF images of illuminations.

### 3.2. Annotation of Written Supports in Illuminations

The presence of books is an essential aspect of liturgical song representations. Although in the Middle Ages, songs were mostly memorized since they were repeated every day in ceremonies and mass, some songs required different texts depending on the days and times of the liturgical year. Therefore, due to the increase in the repertoire and the development of polyphony, it was necessary to create written supports. Examples of written supports are missals and graduals. The size of written supports may vary from a hand-held book to a large codex requiring a lectern, around which the singers would gather, in the middle of the choir. Written supports coud be found in the hands or knees, tables, altars, lecterns, palace hall, or gardens.

Another type of written support for singing performances is phylacteries and sheets (with or without musical notation). There are well-known phrases that are always sung, such as “Ave Maria Gratia plena”, “Cantate Domino”, or “Gloria in Excelsis Deo”. When these phrases are found in phylacteries or the texts around illuminations, they indicate a singing performance.

**Figure 2:**
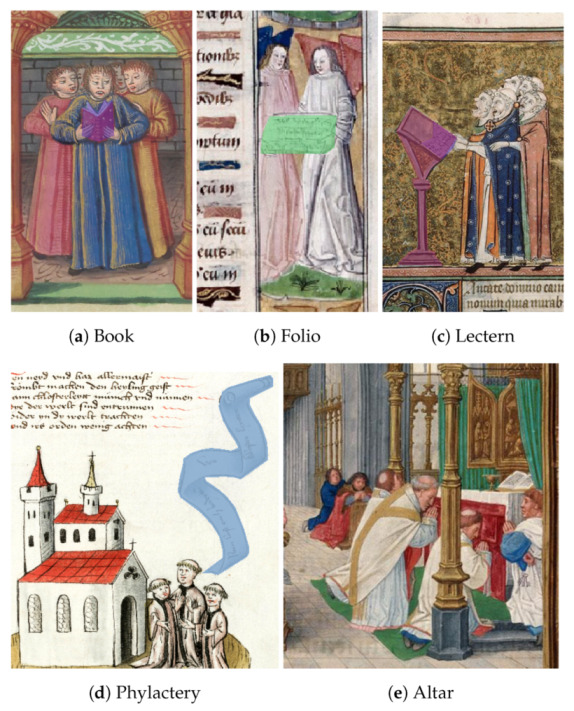
Examples of object annotations.

Therefore, in a previously selected dataset of IIIF images selected manually by experts containing singing performances, we annotated the following classes as described in Table 1:

1. Phylactery: a medieval speech scroll, which contains or depicts speech, song, or other sounds (for example, Figure 2d (Bodmer ms. 91 f. 39r: http://www.e-codices.unifr.ch/loris/fmb/fmb-cb-0091/fmb-cb-0091_039r.jp2/full/full/0/default/jpg, accessed on 1 November 2021)).
2. Folio: a thin and flat surface that can be used for writing or drawing. (Figure 2b (Abbeville ms. 016 f. 15: https://iiif.irht.cnrs.fr/iiif/France/Abbeville/B800016201/DEPOT/IRHT_106357_2/1000,500,800,1500/full/0/default.jpg, accessed on 1 November 2021)).
3. Book: a collection of sheets bound together containing printed or written texts, pictures, etc. (Figure 2a (BnF ms. fr. 166 f. 119v: https://gallica.bnf.fr/iiif/ark:/12148/btv1b105325870/f252/1500,3100,1000,1200/full/0/native.jpg, accessed on 1 November 2021)).
4. Altar: a sacred table used for ritual sacrifice or offerings in a religious building. (Figure 2e (Initiale ms. 3028 f.082 http://initiale.irht.cnrs.fr/decor/58000, accessed on 1 November 2021)).
5. Lectern: a reading desk with a slanted top, on which books are placed for reading aloud. (Figure 2c (Avignon ms. 0121, f. 162v: https://bvmm.irht.cnrs.fr/consult/consult.php?mode=ecran&reproductionId=15460&VUE_ID=1393519, accessed on 1 November 2021)).

## 4. Methodology

In this section we describe our novel method for few-shot object detection based on a bi-training approach. We start by first describing the context of few-shot object detection and the type of input we work with; then we describe in detail the different steps and loss functions for our bi-training method. We finish this section with a description of the contributions provided in this article.

We use the same description of few-shot object detection as the settings introduced in [6]. We have a set of base classes $C_{b}$, which contain a sufficient number of samples (sufficiency depends on the model and the pretraining and the method used for training, which we will investigate in the results section) and a set of novel classes $C_{n}$ that have a low representation in the dataset, with only *K* objects per class (where K is small number, usually around 10; we investigate different values of K in the results section). For the object detection task we need D=(x,y),x∈X,y∈Y such that *x* is the input image and $y=(c_{i},l_{i}),i=1,\cdots ,N$ is the set of annotations per image that is made with the bounding box coordinates and the class of each object such that the set of classes is the union of base classes and novel classes. For our dataset, we use a different ratio of novel/base classes and different thresholds for the definition of a novel and base class.

To evaluate our method for few-shot object detection, we used a test set that contained a combination of both novel and base classes, with the final goal of optimizing the performance of the model on both sets, which we quantified using the mean average precision (mAP) metric, which combines the results on all classes.

### 4.1. Bi-Training Few-Shot Object Detection

We describe our bi-training few-shot object detection (BiT) method in this section. We demonstrate that our method is model-agnostic and can work with a variety of model architectures, allowing it to be used on any pretrained object detector, fine-tuning it for enhanced performance. We chose to use the faster RCNN approach with a region proposal network (RPN) backbone as the representative of region-based convolutional neural networks, and to use Yolov4 as the representative of the You Only Look Once family and the Swin T model as a representative of the vision transformer family. Intuitively, our method aims at treating the object detector as a black box but makes a few assumptions about it. The first assumption is the existence of an object proposal sub-network (for most two-stage models this exists easily but for Yolo it is represented as a model with only the “objectness score calculations” without performing the object classification, making it a class-agnostic sub-model). The second assumption is the existence of an object classifier, which is the case for all of our models. Our method operates on two training steps described in Algorithm 1, which are:
**Algorithm 1:** Bi-Training Few-Shot Object Detection
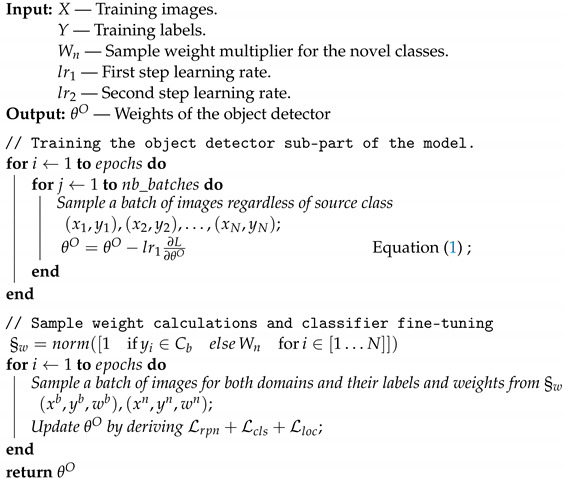


#### 4.1.1. Total Model Improvement

The first step in our method is to fine-tune the object detector (not the classifier) on the whole dataset, not only the base classes, to make sure the model is able to propose objects for classification for all classes, especially if the novel classes are not very similar in shape and style to the base classes. This step can be applied to all object detection models since they are all either two-step models or YOLO-based, where the classification and object proposal happen in the same step, and this can be achieved by using a weighted combination of both losses, where the weight of the classification at this step is 0. The joint loss would look like this:

(1) $$L=L_{rpn}+0\times L_{cls}+L_{loc},$$

such that $L_{rpn}$ represents the object proposal loss function applied on the RPN (or the object scoreness cross entropy loss for YOLO-based models), which mainly used to refine the anchors without touching the feature extractor (the backbone remains fixed as our dataset does not have enough samples to effectively retrain the whole model). $L_{cls}$ is the object classification cross entropy loss and in this stage we do not train it; we try to ignore its effect because the object proposal step at this stage still is not good enough to propose enough samples of the novel classes, leading to an even bigger class imbalance problem for the classifier. Finally, $L_{loc}$ is the smoothed L1 loss used to train the box regressor.

#### 4.1.2. Classifier Fine-Tuning

The second step of our model treats the object detector as a whole as a two-step model, in which the first step is the object proposal and the second is the classification and bounding box regression. In our first step we fine-tuned the object proposal model and now, for the fine-tuning of the classifier and bounding box regressor, we will treat it as we would another CNN model used for classification. This simplification works very well on all object detector models, regardless of whether they are one-step models (Yolo) or two-step models (RCNN). The fine-tuning is targeted only at the last layer of the bounding box regressor and the classifier (if they are seperate layers, as in the RCNN family, they are fine tuned separately, and if they are in the same layer, as in YOLO, they are done together). We fine-tune both base classes and novel classes but we assign a higher sample weight to the novel samples, forcing the model to perform better with the novel samples. We decrease the learning rate in this stage compared to the first stage, allowing the model to train slower and not to change drastically in order to fit the novel classes and abandon all results on the base classes.

## 5. Results

In this section, we conduct extensive benchmarking of several object detectors on the task of few-shot object detection based on two approaches. The first one aims at measuring the influence of reducing the overall number of samples for both base classes and novel classes on the performance ofthe model in total. The second experiment was conducted to see how the change in the number of samples of the lowest novel class influenced its own average precision. In our paper, we focus only on deep learning models instead of machine learning techniques, mainly because our dataset contains a large variety in forms shapes, sizes and artistic representations within the same group of objects. Nevertheless, our dataset has a small sample count for each class, making it a very difficult task for machine learning models which do not leverage pre-training and transfer learning. For the sake of a better evaluation, we trained a histogram of oriented gradients, followed by a support vector machine classifier and a sliding bag of visual words. The results for the Bag Of Visual Words BOVW model were almost zero regardless of the fine tuning, but the Histogram of Oriented Gradients HOG model had a high detection rate (74/138–80% training, 20% testing) but a very low precision since the model confused many of the other parts of the image as an object, dropping the f1-score to 0.239. The threshold of the intersection over union used for the experiment was 50% to increase the model’s results.

We used multiple models for benchmarking, mainly the yolov4 (m/s), mask RCNN (faster RCNN/Mask RCNN), and ViT (Swin-t/ViT) models. Each one of these models was trained fully on the dataset to provide us with the upper bound estimation of the baseline for few-shot object detection, which expresses the full object detection capabilities of the model. We also use a lower bound baseline to prove the effectiveness of our method for few-shot object detection by training the models on the few-shot data directly, without adding any emphasis on the novel classes. For the sake of the experiments in the rest of this section, we selected two base classes, which were livre (book), and phylactère (phylactery), mainly because they were highly represented in our dataset, with 338 objects for book and 204 for phylactery. The novel classes are represented in the rest of the object classes, specifically: lutrin (lectern), autel (altar), feuillet (leaflet), texte chanté (sung text), which were much less represented in the dataset, with lectern having 87 samples, whereas the others had between 20 to 30 samples each. Although many methods have been proposed for hyper-parameter tuning, such as Bayesian-optimized bidirectional LSTM [35] and Google Vizier [36], we chose to hyper-parameter-tune our models using the default hyper-parameters of each backbone or using grid-search cross validation, as described in hyperparameter optimization [37], with $n_{epochs}=2000\times n_{c}$ and $n_{c}=n_{b}+n_{n}$ as the total numbers of classes (base and noval), whereas the learning rates, for example, were set to [0.0001, 0.00025, 0.001, 0.01, 0.00001], and through grid search CV we found that we obtained the most optimal results by using $lr_{1}=0.00025$ and $lr_{2}=0.00001$.

### 5.1. Global Few-Shot Object Detection Benchmark

We provide the average AP50 of the models on the whole dataset of medieval singing images with different distribution percentages of data (100%, 80%, 50%). These percentages applied to each object class individually to keep the same ratio of classes in the dataset. The goal of this evaluation was to see how the model’s performances changes on novel classes and on base classes.

**Figure 3:**
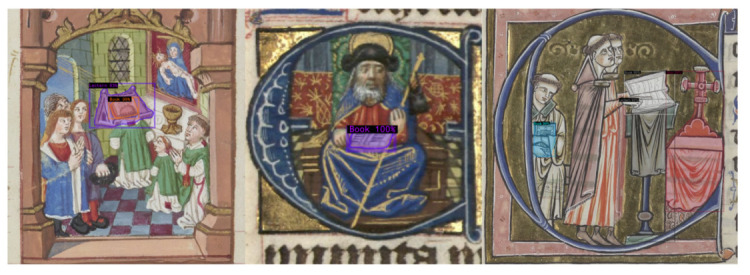
Inference of the Mask RCNN architecture for few-shot instance segmentation on our medieval singing dataset.

The data were split into training and testing data, following different ratios, 90% 10% for the base classes and 60% 40% for the novel classes, allowing a relatively good amount of objects for novel classes to evaluate and extract meaningful information and ranking between models. As the number of testing samples for the novel classes still remains too small to be statically significant to extract useful interpretations, we report the median results of the models over five repetitions of the same experiment but with random train test splits, allowing for more stable estimations of the performances of each model and for the effectiveness of our few-shot object detection method, and Figure 3 shows some inference results on our dataset for this.

Table 2 shows the mean average precision values of the different models on different versions of our dataset. We can see from the results of the upper boundary with 100% data that the order of models was as follows—yolov4-s was the best for our dataset, providing good performance for both base and novel classes, followed by mask RCNN and yolov4-m, whereas the Swin-t model provided good results on the base classes and average results for the novel classes. The worst model for our dataset was ViT, which requires a lot of data to train a good model and is thus not suitable for few-shot object detection tasks and cultural heritage applications in general. We can also clearly see that our model always improves over the lower boundary of transfer learning when it is used in isolation for all models and all versions of the dataset, proving the effectiveness of our simple yet effective method. Our model works best for the Mask RCNN model, which is the most compatible with our idea and assumptions, showing a performance for 80% similar to 100% and for 50% similar to the LB for 80%.

### 5.2. Worst-Case Few-Shot Object Detection Benchmark

In this section we evaluated the performance of the different object detection models on the task of few-shot object detection while changing the number of samples in the smallest class and keeping the others full. This gives us an estimation of the worst-case performance for a specific class by any of these different object detectors, allowing for a better benchmarking and better decision-making when choosing a model for different application areas.

Table 3 shows the results of the different models on the different states of our dataset, especially for the lowest represented class. We repeated each experiment five times and each time we randomly selected a class and set its instances for training to the chosen number for the experiment, and we report the mean average precision of the model over all the experiments.

The previously described setup allows us to obtain a good estimation of what would be the worst results obtained by each model in the task of few-shot object detection. We can see from the results that the ordering is still the same, with yolov4-s still taking the lead, followed by Mask RCNN. Our method also improved the performances over the transfer learning baseline, showing the effectiveness of our method. We can also see that attention-based methods are very useful and are indeed the current state-of-the-art for object detection but they still require a lot more data to train a good model than all other previous methods such as Yolos and different versions of RCNN.

## 6. Conclusions

We have presented a new manually-annotated object detection and instance segmentation dataset for medieval musicological studies and a non-intrusive black-box few-shot object detection method. Our Medieval Musicological Studies Dataset (MMSD) provides a good challenge for transfer learning and few-shot object detection methods. Our dataset is more challenging than the current benchmarks used for evaluation because of the large variety of styles and the significant differences between the artistic representations of the objects and their real shapes. Our new and simple few-shot object detection method integrates seamlessly with all state-of-the-art models for object detection, such as YOLO-based, RCNN-based, and attention-based methods, and provides a significant performance increase over traditional transfer learning methods, yet remains very limited in extreme cases where sample counts are very small. We also concluded that attention-based models are very powerful, but they require more training data, unlike models such as YOLOv4 s and YOLOv5 v6.

## Figures and Tables

**Table 1:** Distribution of objects in our dataset across different classes.

Class	Counts
Book	338
Phylactery	204
Lectern	87
Altar	37
Folio	27
TOTAL	693

**Table 2:** Average Precision evaluation of different state-of-the-art models for object detection using our newly proposed dataset. LB refers to the lower bound baseline, which is transfer learning with the same data, and UB refers to upper bound baseline, which is a transfer with the full dataset.

		Percentage									
		100% UB		80% Ours		80% LB		50% Ours		50% LB	
		Base	Novel	Base	Novel	Base	Novel	Base	Novel	Base	Novel
Yolov4	s	83.62	81.86	61.88	62.24	50.24	55.66	54.78	48.57	52.85	51.30
	m	72.86	77.19	65.27	58.87	58.28	58.66	36.94	52.54	33.76	36.77
RCNN	Faster	68.90	72.04	61.55	58.44	52.39	54.54	46.77	47.19	42.04	37.08
	Mask	79.13	77.472	71.31	62.49	59.77	62.17	55.58	57.03	49.15	54.03
ViT	ViT	57.13	54.96	45.27	42.72	36.84	50.12	33.05	35.55	28.53	23.26
	Swin-t	72.08	57.49	65.18	45.35	48.75	45.25	44.30	46.77	38.06	44.49

**Table 3:** Average precision evaluation of different state-of-the-art models for object detection using our newly proposed dataset. LB refers to the lower bound baseline, which is transfer learning with the same data, and UB refers to the upper bound baseline, which is a transfer with the full dataset.

		Lowest Novel Count				
		Full UB	10 Ours	10 LB	5 Ours	5 LB
Yolov4	s	76.15	59.78	52.13	49.71	43.79
	m	71.89	53.17	54.40	48.86	31.82
RCNN	Faster	68.17	52.85	53.35	42.08	38.57
	Mask	72.65	57.92	57.06	51.14	48.60
ViT	ViT	52.69	46.18	41.92	35.97	24.05
	Swin-t	53.61	47.99	40.20	41.88	39.28

## Data Availability

The dataset will be shared on a public GitHub repository with a detailed description of the images and their contents as part of a publication from the musicology labof Sorbonne University.

## References

[1] Bekkouch I.E.I., Eyharabide V., Billiet F. Dual Training for Transfer Learning: Application on Medieval Studies. Proceedings of the 2021 International Joint Conference on Neural Networks (IJCNN).

[2] Bekkouch I.E.I., Constantin N.D., Eyharabide V., Billiet F., Arai K. (2022). Adversarial Domain Adaptation for Medieval Instrument Recognition. Intelligent Systems and Applications.

[3] Bekkouch I.E.I., Aidinovich T., Vrtovec T., Kuleev R., Ibragimov B., Išgum I., Landman B.A. (2021). Multi-agent shape models for hip landmark detection in MR scans. Medical Imaging 2021: Image Processing.

[4] Bekkouch I.E.I., Nicolae D.C., Khan A., Kazmi S.M.A., Khattak A.M., Ibragimov B. (2021). Adversarial Reconstruction Loss for Domain Generalization. IEEE Access.

[5] Wu B., Xu C., Dai X., Wan A., Zhang P., Yan Z., Tomizuka M., Gonzalez J., Keutzer K., Vajda P. (2020). Visual Transformers: Token-based Image Representation and Processing for Computer Vision. arXiv.

[6] Kang B., Liu Z., Wang X., Yu F., Feng J., Darrell T. Few-shot object detection via feature reweighting. Proceedings of the IEEE/CVF International Conference on Computer Vision.

[7] Wang X., Huang T.E., Darrell T., Gonzalez J.E., Yu F. (2020). Frustratingly Simple Few-Shot Object Detection. arXiv.

[8] Wang Y., Yao Q. (2019). Few-Shot Learning: A Survey. arXiv.

[9] Vanschoren J. (2019). Meta-learning. Automated Machine Learning.

[10] Batanina E., Bekkouch I.E.I., Youssry Y., Khan A., Khattak A.M., Bortnikov M. Domain Adaptation for Car Accident Detection in Videos. Proceedings of the 2019 Ninth International Conference on Image Processing Theory, Tools and Applications (IPTA).

[11] Bochkovskiy A., Wang C.Y., Liao H.Y.M. (2020). YOLOv4: Optimal Speed and Accuracy of Object Detection. arXiv.

[12] Ren S., He K., Girshick R.B., Sun J. (2015). Faster R-CNN: Towards Real-Time Object Detection with Region Proposal Networks. Adv. Neural Inf. Process. Syst..

[13] Liu Z., Lin Y., Cao Y., Hu H., Wei Y., Zhang Z., Lin S., Guo B. Swin Transformer: Hierarchical Vision Transformer Using Shifted Windows. Proceedings of the IEEE/CVF International Conference on Computer Vision (ICCV).

[14] Girshick R. Fast r-cnn. Proceedings of the IEEE International Conference on Computer Vision.

[15] He K., Gkioxari G., Dollár P., Girshick R. Mask r-cnn. Proceedings of the IEEE International Conference on COMPUTER Vision.

[16] Girshick R., Donahue J., Darrell T., Malik J. Rich feature hierarchies for accurate object detection and semantic segmentation. Proceedings of the IEEE Conference on Computer Vision and Pattern Recognition.

[17] Farhadi A., Redmon J. (2018). Yolov3: An incremental improvement. Computer Vision and Pattern Recognition.

[18] Yakovlev K., Bekkouch I.E.I., Khan A.M., Khattak A.M. Abstraction-Based Outlier Detection for Image Data. Proceedings of the SAI Intelligent Systems Conference.

[19] Rivera A.R., Khan A., Bekkouch I.E.I., Sheikh T.S. (2020). Anomaly Detection Based on Zero-Shot Outlier Synthesis and Hierarchical Feature Distillation. IEEE Trans. Neural Netw. Learn. Syst..

[20] Ibrahim B.I., Nicolae D.C., Khan A., Ali S.I., Khattak A. (2020). VAE-GAN Based Zero-Shot Outlier Detection. Proceedings of the 2020 4th International Symposium on Computer Science and Intelligent Control (ISCSIC 2020).

[21] Choi H.T., Lee H.J., Kang H., Yu S., Park H.H. (2021). SSD-EMB: An Improved SSD Using Enhanced Feature Map Block for Object Detection. Sensors.

[22] Afif M., Ayachi R., Said Y., Pissaloux E., Atri M. (2020). An evaluation of retinanet on indoor object detection for blind and visually impaired persons assistance navigation. Neural Process. Lett..

[23] He K., Zhang X., Ren S., Sun J. (2015). Spatial pyramid pooling in deep convolutional networks for visual recognition. IEEE Trans. Pattern Anal. Mach. Intell..

[24] Touvron H., Cord M., Douze M., Massa F., Sablayrolles A., Jegou H., Meila M., Zhang T. (2021). Training data-efficient image transformers & distillation through attention. Proceedings of the 38th International Conference on Machine Learning, Online, 18–24 July 2021.

[25] Li K., Huang Z., Cheng Y.C., Lee C.H. A maximal figure-of-merit learning approach to maximizing mean average precision with deep neural network based classifiers. Proceedings of the 2014 IEEE International Conference on Acoustics, Speech and Signal Processing (ICASSP).

[26] Chen W., Liu Y., Kira Z., Wang Y.F., Huang J. (2019). A Closer Look at Few-shot Classification. arXiv.

[27] Finn C., Abbeel P., Levine S. Model-agnostic meta-learning for fast adaptation of deep networks. Proceedings of the International Conference on Machine Learning.

[28] Eyharabide V., Bekkouch I.E.I., Constantin N.D. (2021). Knowledge Graph Embedding-Based Domain Adaptation for Musical Instrument Recognition. Computers.

[29] Elgammal A., Kang Y., Den Leeuw M. Picasso, matisse, or a fake? Automated analysis of drawings at the stroke level for attribution and authentication. Proceedings of the Thirty-Second AAAI Conference on Artificial Intelligence.

[30] Xu Z., Wilber M., Fang C., Hertzmann A., Jin H. (2018). Learning from multi-domain artistic images for arbitrary style transfer. arXiv.

[31] Sabatelli M., Kestemont M., Daelemans W., Geurts P. Deep transfer learning for art classification problems. Proceedings of the European Conference on Computer Vision (ECCV) Workshops.

[32] Sharafi S., Fouladvand S., Simpson I., Alvarez J.A.B. (2016). Application of pattern recognition in detection of buried archaeological sites based on analysing environmental variables, Khorramabad Plain, West Iran. J. Archaeol. Sci. Rep..

[33] Lambers K. (2019). Learning to look at LiDAR: The use of R-CNN in the automated detection of archaeological objects in LiDAR data from the Netherlands. J. Comput. Appl. Archaeol..

[34] Bekkouch I.E.I., Youssry Y., Gafarov R., Khan A., Khattak A.M. (2019). Triplet Loss Network for Unsupervised Domain Adaptation. Algorithms.

[35] Kaselimi M., Doulamis N., Doulamis A., Voulodimos A., Protopapadakis E. Bayesian-optimized Bidirectional LSTM Regression Model for Non-intrusive Load Monitoring. Proceedings of the ICASSP 2019–2019 IEEE International Conference on Acoustics, Speech and Signal Processing (ICASSP).

[36] Golovin D., Solnik B., Moitra S., Kochanski G., Karro J., Sculley D. Google vizier: A service for black-box optimization. Proceedings of the 23rd ACM SIGKDD International Conference on Knowledge Discovery and Data Mining.

[37] Feurer M., Hutter F. (2019). Hyperparameter optimization. Automated Machine Learning.

